# Chemical and biochemical bleaching of oat hulls: The effect of hydrogen peroxide, laccase, xylanase and sonication on optical properties and chemical composition

**DOI:** 10.1016/j.btre.2021.e00624

**Published:** 2021-04-29

**Authors:** Eva Schmitz, Juanita Francis, Katarina Gutke, Eva Nordberg Karlsson, Patrick Adlercreutz, Magnus Paulsson

**Affiliations:** a Department of Chemistry, Division of Biotechnology, Lund University, PO-Box 124, 22100 Lund, Sweden; b Nouryon, 44580 Bohus, Sweden

**Keywords:** Oat hulls, Chemical bleaching, Hydrogen peroxide, Biobleaching, Enzymes, Dietary fibre

## Abstract

Oat hulls are an excellent dietary fibre source for food supplements due to their rich lignocellulose composition as well as their great abundance as low-value agricultural side stream. For the production of white fibre supplements, a mild, but effective bleaching of the hulls is required. Chemical bleaching with hydrogen peroxide and sodium hydroxide was here found to be a suitable method increasing the CIE L* value (corresponds to a lightness value) above 85. The developed method is mild, retaining the hull’s chemical composition. Only a minor decrease in coniferaldehyde structures upon bleaching was detected. Colour and chemical variabilities of oat hulls from different growth seasons did not influence the required bleaching conditions to achieve the desired optical properties. The inclusion of biochemical bleaching steps utilizing the xylanase Pentopan Mono BG, the laccase NS51003 and sonication was industrially not feasible as they could not reduce the required amount of subsequently applied bleaching chemicals significantly.

## Highlights

• Alkaline hydrogen peroxide is a mild and robust bleaching method for oat hulls.
• The lignocellulose content is not altered during alkaline H_2_O_2_ bleaching.
• Neither phenolic acids nor coniferaldehyde structures are removed during bleaching.
• Laccase, xylanase and sonication are not suitable for biobleaching of oat hulls.

## Graphical abstract

**Figure:**
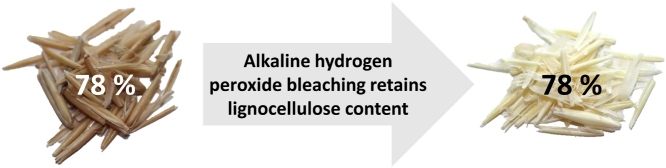

## 1 Introduction

In recent years, the consumer interest for healthier food products has increased, including fibre-enriched products. Oat hulls are an excellent source for additional dietary fibre due to their rich lignocellulose composition as well as their great abundance as low-value byproduct from oat production. Their lignocellulose content can reach up to 84 % and the hemicellulose fraction up to 35 % depending on the growth conditions [1]. This makes the oat hull composition superior to wheat straw, which is currently commonly used as fibre supplement. Wheat straw contains a similarly large lignocellulose fraction, however only 23–30 % is hemicellulose [2]. The hemicellulose from cereals is of particular interest as a fibre supplement as it can be either added as insoluble fibre to reduce hunger feelings for an increased amount of time after consumption [3] or broken down into smaller soluble fragments with potentially prebiotic effects in the gut [4]. However, there is an important drawback of utilizing oat hulls as fibre supplements. Oat hulls are naturally dark in colour, which alters the optical appearance of the end products, such as pasta or bread. White fibres are generally preferred as they can easily be incorporated in food formulations without altering the optical properties. Therefore, bleaching of oat hulls is a required processing step for their use in food products.

Bleaching can be performed either with chemical or biochemical methods or a combination thereof. Traditional chemical bleaching typically employed elemental chlorine, which made the process very effective and economical, but environmentally unfriendly. To counteract this negative aspect, modern chemical bleaching follows either totally chlorine-free methods or elemental chlorine free methods, which use chlorine chemicals such as chlorine dioxide or hypochlorite. Both methods utilize oxygen based bleaching agents, such as elemental oxygen, hydrogen peroxide (H_2_O_2_) or ozone [5]. The inclusion of biochemical methods could also lower the negative impact of bleaching on the environment by reducing the consumption of the required bleaching chemicals to achieve similar or better optical properties. These methods could, therefore, contribute to the achievement of the sustainable development goal 12 discussing responsible consumption and production [6]. A very promising candidate is the enzyme laccase. Laccases are multicopper oxidases, which are capable of oxidizing phenols and even non-phenolic lignin units, when combined with an oxidation mediator. During the laccase reaction with its substrate a phenoxy radical is formed, which can depolymerise lignin and hence increase the brightness of the material. However, the action of the phenoxy radical could also result in counterproductive lignin repolymerization as well as their conversion into chromophoric conjugated carbonyl structures, such as quinones, which darken the material instead. These chromophoric structures can be removed by alkaline or oxidative chemical bleaching stages with a lower chemical loading than if only chemical bleaching had been performed [5]. Therefore, a combination of laccase and chemical bleaching is very attractive.

Previous studies on both wood kraft pulp and lignocellulosic material from agricultural sources have shown that the use of xylanase alone or in combination with laccase can reduce the need for bleaching chemicals [[7], [8], [9]]. Xylanases are capable of separating the lignin carbohydrate complexes, which makes the material more accessible to the bleaching chemicals. In one case, the combined treatment of xylanase and laccase on kraft pulp made a reduction of the bleaching chemical sodium hypochlorite of up to 42 % possible [9]. A similar loosening of the lignin carbohydrate complexes was observed by Sun and colleagues [10], when extracting hemicellulose from sugarcane bagasse via ultrasonication. Therefore, sonication could be another potential processing step to reduce the required amount of both chemical and biochemical agents for bleaching.

Only chemical bleaching has so far been tested on oat hulls utilizing either alkaline (NaOH) hydrogen peroxide extrusion [[11], [12], [13]] or peracetic acid [14,15]. Alkaline hydrogen peroxide extrusion caused an increase of the oat hull’s L* value, which measures the grey-scale, of 15 % (from 44.8–51.4) [11] and 23 % (from 46.8–53.5) [12]. However, neither of the studies aimed at increasing the optical properties of the oat hulls, but rather their modification for fibre functionalization or isolation of nanofibrillated cellulose. Therefore, the bleaching methods were not optimized towards this parameter. The alkaline hydrogen peroxide bleached hulls were, however, also tested for their suitability as fibre supplement in a food product, i.e. cookies, regarding their physical and sensory characteristics [16]. A very high acceptance level of 91 % among consumers was reached, indicating the suitability of utilizing alkaline hydrogen peroxide as bleaching agent.

The present study assesses the chemical and biochemical bleachability, including sonication, of oat hulls and evaluates the changes imposed by the developed process on their fibre composition. The main objective is to find a suitable bleaching method yielding good optical properties for use in the food industry for the production of white fibre supplements.

## 2 Materials and methods

### 2.1 Raw materials, enzymes and chemicals

Four different oat hull batches were supplied by Lantmännen ek. för. All batches were a mixture of Kerstin and Galant varieties grown in central Sweden (Mälaren Valley, with minorities coming from Östergötland (English exonym East Gothland) and Västergötland (English exonym West Gothland)) in different years. All seeds were obtained from SW-Seed (Sweden). As the chemical composition of oat hulls can vary considerably depending on the environmental conditions during the growth season [1], the darkness of the batches differed visibly. A more detailed description of the batches is given in Table 1, Table 2. The hulls were industrially separated from the grains utilizing a Bühler BSSA stratopact HKE50HP-Ex peeler (Höflinger Millingsystems) and milled for the biochemical bleaching study using an industrial size hammer mill with a milling capacity of 1 t/h located at the Lantmännen factory in Järna.

**Table 1:** Description of oat hull batches used in this study. The data were taken from our previous study [1] and the methods described below. The copper, iron and manganese content before and after milling (in brackets) is given.

Batch	Harvest year	Lignocellulose content [%]^1^	Starch content [%]^1^	Ferulic acid content [μg/g]^1^	Cu [μg/g]	Fe [μg/g]	Mn [μg/g]
Swe16	2016	74.5 ± 1.0	8.5 ± 0.4	2596 ± 40	1.3	11	27
Swe17	2017	82.7 ± 0.4	2.5 ± 0.6	2319 ± 111	1.7 (1.4)	18 (16)	36 (31)
Swe18	2018	62.3 ± 0.5	16.3 ± 1.9	1339 ± 41	1.4 (3.3)^2^	17 (76)^3^	27 (52)^2^
Swe19	2019	71.7 ± 0.4	12.1 ± 0.5	3327 ± 222	2.4 (2.4)	24 (45)^3^	40 (42)

1 Analysis performed on milled oat hulls.

2 The higher content in the milled oat hulls indicates that the milled and unmilled hulls are from different batches as this increase is not seen for Swe17 and Swe19.

3 Batches Swe18 and Swe19 were milled with industrial milling equipment, while Swe17 was milled in a lab mill. The higher Fe content in batches Swe18 and Swe19 is likely due to contamination from the industrial milling equipment since the Fe content of the different unmilled oat hull batches was in the range 11−24 μg/g.

**Table 2:** Description of the optical properties of the unmilled oat hull batches used in this study. The optical properties are described using the three-dimensional colour system CIE L*, a*, b*, which consists of a grey scale axis (L*), a green-red axis (a*), and a blue-yellow axis (b*).

Batch	Harvest year	Brightness [%]	L*	a*	b*
Swe16	2016	22.4	66.3	3.0	21.2
Swe17	2017	22.4	64.6	2.4	18.1
Swe18	2018	27.0	70.7	2.9	21.0
Swe19	2019	25.9	68.2	2.3	18.4

The enzyme α-amylase from *Bacillus licheniformis* (type XII-A) was obtained from Sigma-Aldrich for starch removal. For biochemical bleaching, the laccase NS51003 and the glycoside hydrolase family 11 xylanase Pentopan Mono BG from Novozymes were used. For the chemical bleaching, hydrogen peroxide (Eka HP C59, 59 %, Nouryon) and sodium hydroxide (NaOH, 1 M, Scharlau) were used.

### 2.2 Chemical bleaching

**Fig. 1:**
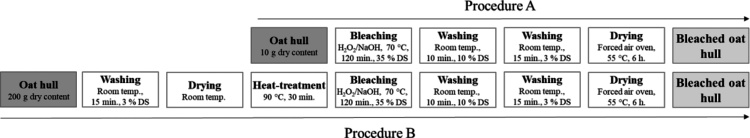
Schematic illustration of the bleaching procedures used in this work. The abbreviation DS stands for dry solids content.

Two different bleaching procedures have been used in this study (see Fig. 1). For the determination of the optimal amount of sodium hydroxide required to reach the highest brightness and L* values at each H_2_O_2_ loading (Swe16–Swe19), for production of a larger amount of bleached material to be milled (Swe17), as well as for the chemical bleaching stage following biochemical bleaching (Swe18, Swe19), the following procedure was used (Procedure A): Hydrogen peroxide (50–150 kg/bone dry ton (bdt)), sodium hydroxide (5–15 kg/bdt) and deionized water were mixed into an oat hull sample (10 g dry weight) by kneading where after the sample (35 % dry solids content) was transferred into a plastic bag that was immersed into a water bath (70 °C) and incubated for 120 min. The chemical loadings and bleaching conditions were chosen based on previous experience (bleaching of wheat bran, unpublished results). The bleaching was stopped by diluting the oat hull sample to a dry solids content of 10 % with deionized water. After 10 min (room temperature), the suspension was dewatered on a Büchner funnel (containing a supporting wire, Monodur, 112 μm) generating a solid fraction and a filtrate (Filtrate I). The bleached oat hull was then washed (3 % dry solids content, 15 min, room temperature) with deionized water, the suspension was dewatered on a Büchner funnel (containing a supporting wire, Monodur, 112 μm) and the solid fraction dried in a forced air oven (Husqvarna Type QW100D) at 55 °C for six hours. The pH and residual H_2_O_2_ content (iodometric titration using 0.05 M sodium thiosulphate from Merck) were determined by analysing Filtrate I. For Swe19, the oat hulls were heat treated (90 °C, 30 min) prior to bleaching to denature enzymes that caused hydrogen peroxide decomposition.

A larger amount of bleached oat hull (Swe19) was prepared in a modified procedure where the bleached oat hull was used for studying the chemical changes that occur during bleaching as well as for evaluating the effect of milling (Procedure B). The following modifications were made to the procedure described above. An unbleached and unmilled oat hull sample (200 g dry weight) was washed in deionized water (3 % dry solids content, 15 min, room temperature) whereafter the suspension was dewatered on a Büchner funnel (containing a supporting wire, Monodur, 112 μm) and the solid fraction dried in room temperature for two days. The dried oat hull was then subjected to a heat treatment (90 °C, 30 min) after which the bleaching procedure was continued as described above. The modifications were done to have a more robust process for industrial implementation, e.g. to be able to handle oat hulls that have been stored for various periods of time and to have a more uniform starting material with low or limited enzymatic activity.

### 2.3 Biochemical bleaching

The bleaching potential of laccase (NS51003), xylanase (Pentopan Mono BG) and sonication on oat hulls was assessed. To establish a suitable protocol for the experiments involving the laccase, the influence of mediator use, oxygen sparging and drying were investigated in a pre-study. All pre-study experiments were carried out on the milled and destarched Swe18 batch. The starch was removed with α-amylase according to the protocol by Sajib and colleagues [17]. Milling before treatment was a necessary process step in order to increase the surface area available for enzymatic attack.

To assess the influence of a mediator on oat hull bleachability, 615 U laccase/g oat hulls (dry weight) were incubated with 0.5, 1 and 3 mM 2,2′-azino-bis(3-ethylbenzothiazoline-6-sulfonic acid) (ABTS; Sigma-Aldrich) at 45 °C for 24 h under constant shaking in a shaking incubator. The reaction was carried out in 0.1 M sodium acetate buffer at pH 4.5 with a 10 % (w/v) solid loading. Subsequently, the samples were centrifuged at 3893 *g* for 20 min and the supernatants discarded. Two cycles of washing with MilliQ water and subsequent centrifuge separation were carried out.

To assess the influence of increased oxygen pressure on oat hull bleachability with laccase, 12 U laccase/g oat hulls (dry weight) were incubated according to the same procedure as above with the exception that air was sparged into the reaction medium for 10 min at the start of the reaction and 4 h later. The washed hulls were dried overnight in a 60 °C oven or freeze dried in a LyphLock 12 lyophilizer (Labconco). Subsequently, the hulls were bleached chemically according to Procedure A described above utilizing chemical loadings of 145 kg/bdt H_2_O_2_ and 10 kg/bdt NaOH.

In order to determine the best conditions for bleaching oat hulls with laccase, xylanase and sonication, a design of experiment study was carried out. The study was planned and statistically analysed utilizing the software MODDE 12.1 (Sartorius Stedim Data Analytics AB) based on an orthogonal (balanced) full factorial design. A detailed description of the variables used is displayed in Table 3. The hulls used for this study were industrially milled Swe19 hulls. In contrast to the oat hulls in the pre-study, the Swe19 hulls were not destarched as their starch content was lower compared to Swe18 hulls (see Table 1) and should therefore not inhibit the enzymatic reactions. The bleaching reactions were carried out successively starting with sonication in Milli-Q water, followed by xylanase treatment in 20 mM sodium phosphate buffer at pH 6.9 and laccase treatment in 0.1 M sodium acetate buffer at pH 4.5, all with a 10 % (w/v) solid loading. For sonication, a Labassco Sonorex RK100H with a frequency of 35 kHz (Bandelin) was used. Both enzymatic reactions were carried out for 24 h at 45 °C under constant shaking. In between treatments the hulls were washed twice with MilliQ water using centrifugation. The final pellets were freeze dried in a LyphLock 12 lyophilizer (Labconco). To assess how the treatment influenced the requirements for subsequently applied bleaching chemicals, the hulls were bleached chemically according to Procedure A described above utilizing 10 kg/bdt NaOH and either 50, 100 or 150 kg/bdt H_2_O_2_.

**Table 3:** Factor settings for the design of experiment study (Swe19 hulls) showing the minimum and maximum values for each factor, which enclose the design space.

Factor	Unit	Minimum	Maximum
Sonication length	min	10	60
Laccase concentration	U/g	6	18
Xylanase concentration	U/g	5	15

### 2.4 Optical properties analyses

**Fig. 2:**
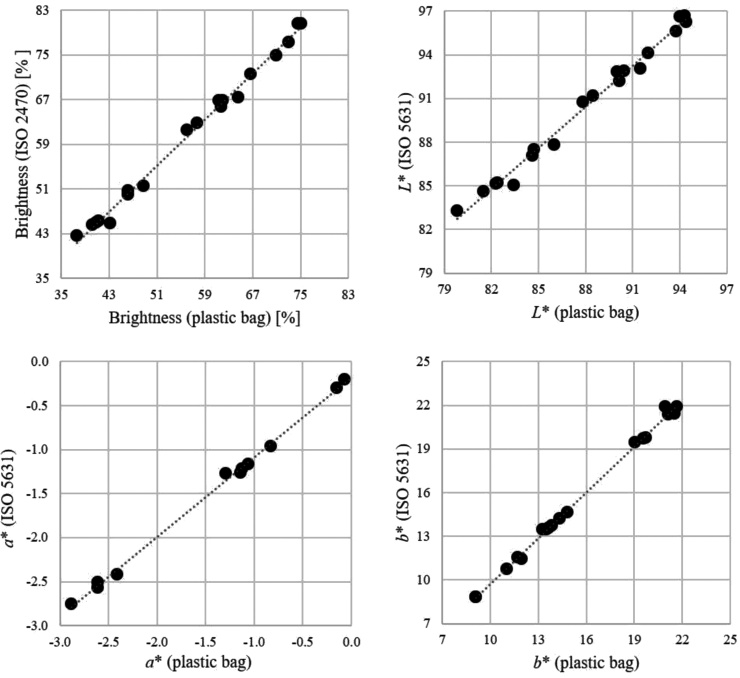
Optical properties (brightness, L*, a* and b*) of wood pulp pad samples produced from unbleached and bleached softwood thermomechanical and kraft pulps measured according to standard procedures (ISO 2470, ISO 5631) and for the corresponding pulp pad samples placed in a plastic bag (the method used in this work).

The optical properties (C, d/0°) of the unbleached as well as bleached oat hulls were analysed using a Technidyne Color Touch X™ spectrophotometer. The dried oat hull samples were disintegrated in a coffee grinder (Krups Type 203B) for 20 s whereafter the gently mechanically treated (still coarse) oat hulls were placed into a plastic bag before determining the optical properties in the spectrophotometer. This method is not according to the standard ISO procedures (ISO 2470; ISO 5631) but was developed to be able to determine the optical properties of particulate matter. In order to determine the correlation between the used method and the standard ISO methods, optical properties of wood pulp pad samples produced from unbleached and bleached softwood thermomechanical and kraft pulps were analysed, using both standard and the modified procedures. The obtained brightness and CIE L* (grey scale axis), a* (green-red axis), and b* (blue-yellow axis) values can be found in Fig. 2. The brightness and L* values were lower when determined using the developed method compared to the ISO method whereas the a* and b* values were less affected. The decrease is about five units for brightness and two to three units for L* when determining the optical properties of samples placed in a plastic bag, i.e. the optical properties of both unbleached and bleached oat hull samples were underestimated in this work.

### 2.5 Structural carbohydrates and lignin content analyses

The lignin, cellulose, hemicellulose and starch content of the 200 g scale unbleached and bleached samples was analysed. Before analysis, the whole hulls were milled in a coffee grinder for one cycle at grind size 8 (Model: BCG820BSSUK; Sage). The lignocellulose fraction was characterized according to the NREL Laboratory Analytical Procedure (NREL/TP-510-42618, 2012). The extracted monosaccharides were identified and quantified utilizing HPAEC-PAD (ICS-5000, Dionex, Thermo Scientific) equipped with a CarboPac PA20 analytical column (150 mm × 3 mm, 6 μm) as well as a respective guard column (30 mm × 3 mm) as previously described by Falck and colleagues [18]. The starch content was determined utilizing the “Total Starch” kit from Megazyme (https://www.megazyme.com/total-starch-assay-kit); method “a” of protocol K-TSTA 09/14 was followed.

### 2.6 Phenolic acid content analysis

The phenolic acids of the 200 g scale unbleached and bleached samples were extracted, separated and quantified according to the HPLC method described by Sajib and colleagues [17]. Before extraction, the whole hulls were milled in a coffee grinder for one cycle at grind size 8 (Model: BCG820BSSUK; Sage). The total phenolic acid content is reported as percentage based on the total phenolic acid content measured in an unbleached reference oat hull sample of the same batch.

### 2.7 Wiesner test

The presence of coniferaldehyde structures in the untreated as well as bleached oat hulls was determined with the Wiesner test [19]. The Wiesner reagent was prepared by dissolving 200 mg phloroglucinol in 8 mL of 20 % ethanol. Subsequently, 2 mL of concentrated hydrochloric acid were added. An oat hull aliquot was immersed in the phloroglucinol−HCl stain for 1 min, after which it was air dried at room temperature overnight. Imaging was performed utilizing a Canon SLR camera.

### 2.8 Metal content analysis

For the detection and quantification of copper, iron and manganese, the oat hulls were first dissolved in nitric acid under wet combustion using a microwave system. Subsequently, the samples were analysed (in duplicate) via inductively coupled plasma optical emission spectroscopy using an iCap 7400 ICP-OES (Thermo Scientific). The report limits for the individual metals in μg/g were as follows: Cu: 0.1; Fe: 0.5; Mn: 0.1.

## 3 Results and discussion

### 3.1 Chemical bleaching

#### 3.1.1 Optimization of alkali to hydrogen peroxide ratio

The chemical bleaching method investigated in this study was based on the combined action of hydrogen peroxide (H_2_O_2_) and sodium hydroxide (NaOH). One stage alkaline hydrogen peroxide bleaching was chosen due to its simplicity and previous experience from bleaching of wheat bran (unpublished results). For each hydrogen peroxide loading there is an optimal alkali loading that gives the highest brightening effect and this loading is influenced by the material to be bleached (e.g. transition metal ion content, amount of acidic groups) and the bleaching conditions employed (time, temperature, dry solids content). Further, the bleaching in this part of the study was performed on unmilled, not destarched oat hulls (Swe16–Swe19) since this makes such a procedure easier to implement in industrial scale (less process steps, ease of dewatering). The prerequisite is that the oat hulls are homogenously bleached, since the dried and bleached hulls are to be milled before mixing into the end products. Two of the oat hull batches (Swe17, Swe19) were therefore milled to a flour after bleaching and the optical properties were determined both before and after milling (see Section 3.1.3).

**Fig. 3:**
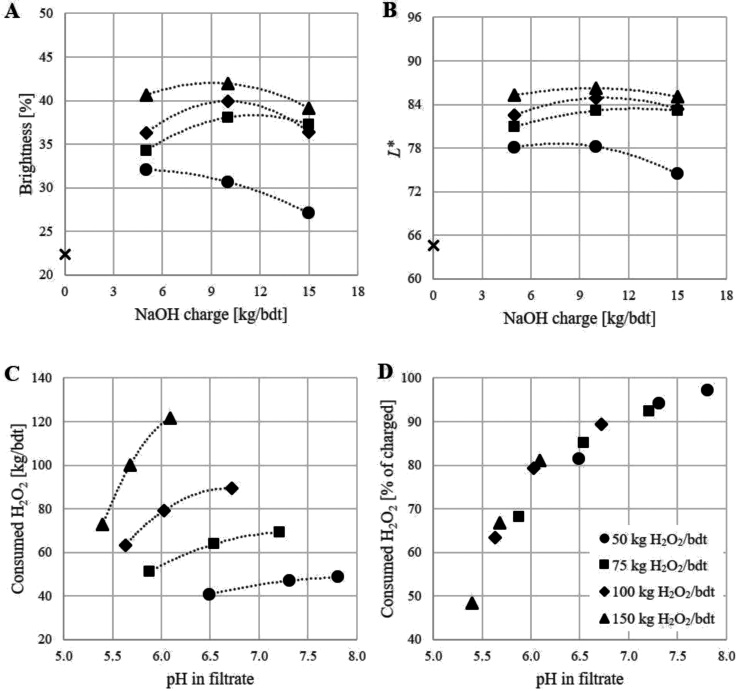
Optimization of alkali loading. The optical properties brightness (A) and L* (B) for unbleached (symbol x) and hydrogen peroxide bleached (filled symbols) unmilled oat hulls (Swe17) as a function of alkali loading. The amount of consumed hydrogen peroxide during bleaching of the hulls is reported as a function of the end pH in the filtrate (C and D).

The bleachability of Swe17 is used to exemplify the importance of optimizing the alkali loading when bleaching with H_2_O_2_. Fig. 3 shows the brightness and L* values for Swe17 at four different H_2_O_2_ loadings (50, 75, 100 and 150 kg/bdt) as a function of the alkali loading. The optimal NaOH loading was found to be 5 kg/bdt for 50 kg H_2_O_2_/bdt and 10 kg NaOH/bdt for the higher hydrogen peroxide loadings. About 65–85 % of the loaded H_2_O_2_ was consumed (higher consumption at the lower H_2_O_2_ loadings) and an increase of up to about 20 and 22 units for brightness and L*, respectively, was possible to achieve. The optical properties were underestimated with the procedure employed in this work (see Materials and methods); the brightness should be about five units higher and the L* value 2–3 units higher than measured. The total yield loss during the bleaching and washing stages was found to be less than 10 %.

#### 3.1.2 Effect of annual variations of oat hulls on bleaching conditions

**Fig. 4:**
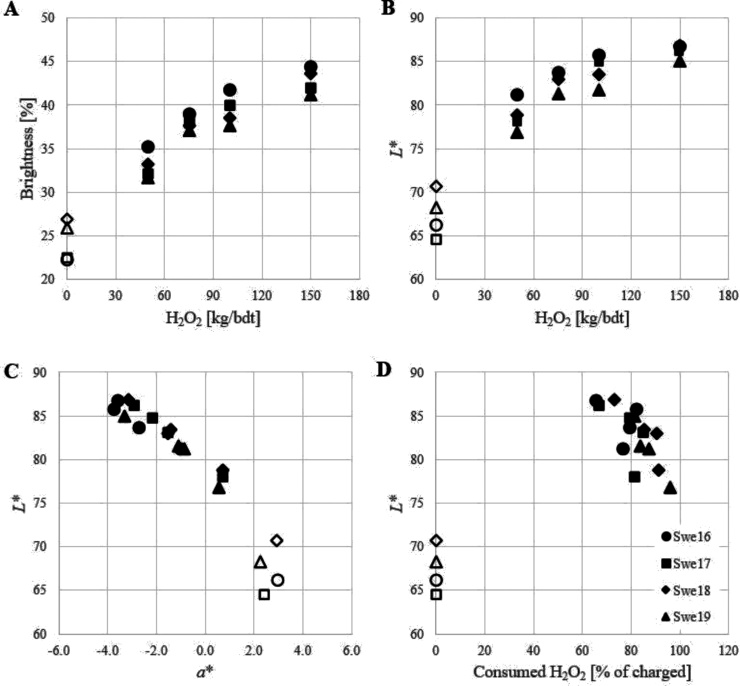
Bleaching of oat hulls from different growth seasons. The optical properties brightness (A) and L* (B) for unbleached (unfilled symbols) and hydrogen peroxide bleached (filled symbols) unmilled oat hulls (Swe16-Swe19) as a function of hydrogen peroxide loading at optimal alkali loading. The L* values are further correlated to the respective a* values (C) as well as the consumed hydrogen peroxide (D). The legend for all four graphs is given in D.

Fig. 4 shows the optical properties for the four unmilled oat hull batches with different growth years (2016–2019) at the optimal alkali loading when bleached with 50−150 kg H_2_O_2_/bdt. The optimal NaOH loading was 5 kg/bdt when charging 50 kg H_2_O_2_/bdt for Swe16-Swe19 and 5−15 kg/bdt for the rest of the H_2_O_2_ loadings (depending on the oat hull batch). Despite the rather large variation in unbleached optical properties (see Table 2) and chemical composition (see Table 1 and reference [1]), the bleaching response was rather similar with a possibility to reach a L* value of above 85 before milling, which corresponds to an 23–33 % increase. Besides increasing the brightness and L* value (lightness), H_2_O_2_ bleaching decreased the a* value (greener) and increased the b* value (more yellow). A previous study on micronized oat hulls reported a similar behaviour for the colour coordinates upon alkaline hydrogen peroxide extrusion [12]. However, the analysis was performed on sheets containing only 10 % oat hulls. Therefore, these results are not directly comparable. The hydrogen peroxide consumption in the present study was higher for Swe18 and Swe19 (75-95 % of loading at optimal alkali loading) compared to Swe16 and Swe17 (65–85 %) which points to a higher H_2_O_2_ decomposition (less H_2_O_2_ available for bleaching) in the former case. The optical properties (especially the L* value) were also lower for Swe18 and Swe19. Hydrogen peroxide decomposition is catalysed by transition metals (especially manganese) but can also be caused by the presence of enzymes (catalase) [20]. The manganese content was, however, similar for Swe16-Swe19 whereas the iron content was higher for Swe19 (Table 1). A heat treatment to deactivate/inhibit enzymatic activity prior to H_2_O_2_ bleaching could improve the robustness of the proposed bleaching process. Removing transition metals using chelating agents (e.g. EDTA) could also be considered even though previous experience with similar materials (wheat bran, unpublished results) and transition metal contents have shown a limited effect of such a treatment.

#### 3.1.3 Effect of milling on optical properties

**Fig. 5:**
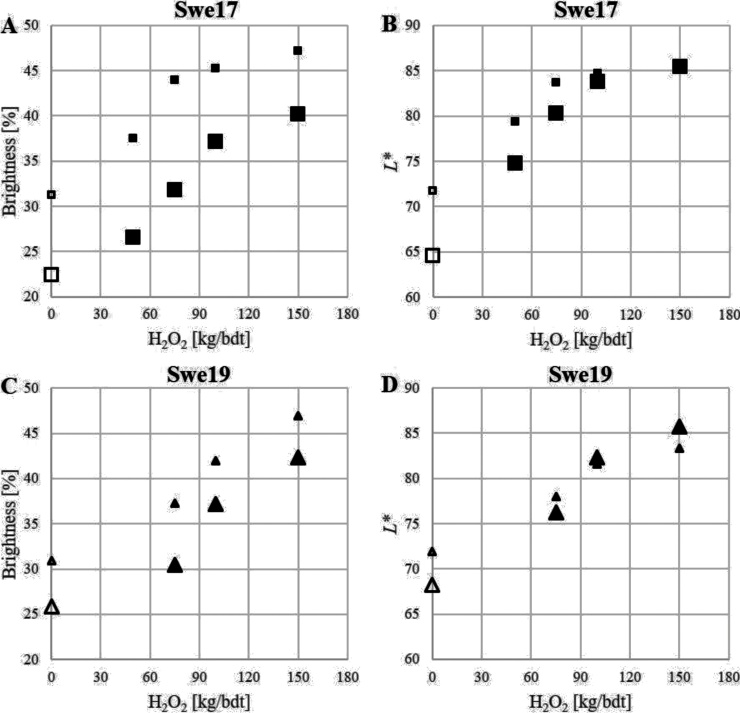
Effects of milling on optical properties. The optical properties brightness (A and C) and L* (B and D) of unbleached (unfilled symbols) and hydrogen peroxide bleached (filled symbols) Swe17 (A and B) and Swe19 (C and D) oat hulls before (large symbols) and after (small symbols) laboratory scale milling in a Perten 3100 mill equipped with a 0.8 mm sieve (Perkin Elmer).

The H_2_O_2_ bleaching described above was performed on unmilled oat hulls. Milling releases new surfaces, hence a reduction of the optical properties would be expected, if the hulls were only bleached on the surface. However, the opposite was observed (see Fig. 5), which implies that the entire material was bleached. The improvement of the optical properties compared to the unmilled hulls is likely to be an effect of an increased light scattering ability. There was, however, a difference between Swe17 and Swe19 oat hull batches where the brightness and L* values increased less for Swe19. It is likely that the two bleaching procedures used could explain this; the mixing of bleaching chemicals into Swe19 (200 g batch) was less effective and it is likely that the bleaching was not as homogeneous as for Swe17 (multiple 10 g batches). Further, the laboratory milling introduced iron (from 18 to 99 μg/g for Swe17 and from 24 to 210 μg/g for Swe19) which could influence the optical properties negatively if coloured iron complexes (with e.g. phenolic compounds) are formed. The increase in brightness was 7–12 and 5–7 units for Swe17 and Swe19, respectively. The increase in the L* value varied but was highest for unbleached and low bleached hulls (Swe17).

#### 3.1.4 Fibre content and composition

**Fig. 6:**
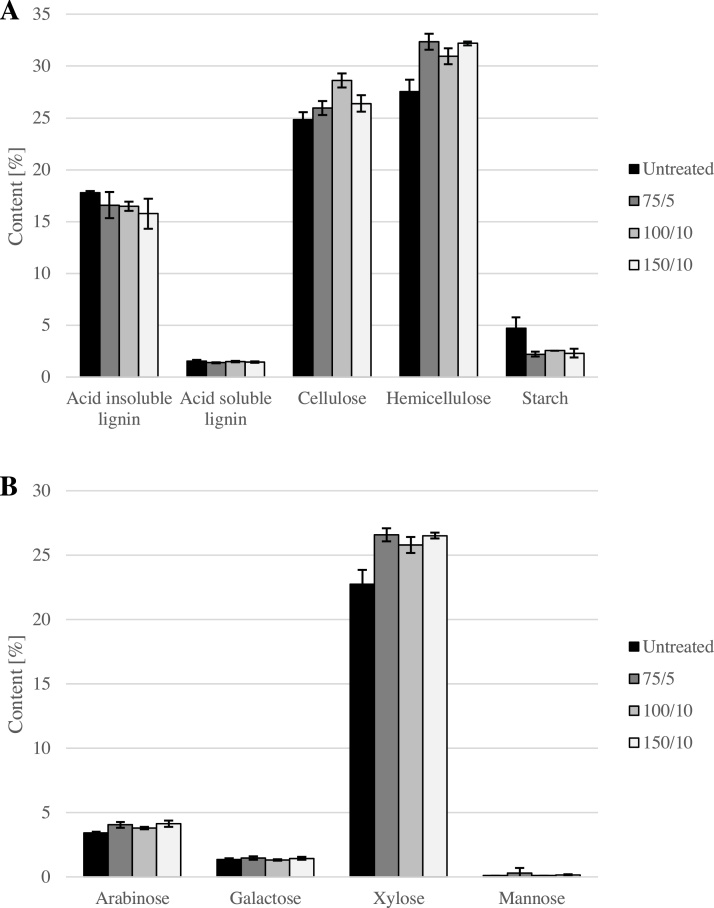
Carbohydrate composition of untreated and bleached Swe19 oat hulls (A) and monosaccharide composition in hemicellulose fraction (B). Bleaching conditions were 75 kg H_2_O_2_/bdt and 5 kg NaOH/bdt (denoted as 75/5 in the graphs), 100 kg H_2_O_2_/bdt and 10 kg NaOH/bdt (100/10) and 150 kg H_2_O_2_/bdt and 10 kg NaOH/bdt (150/10). All analyses were performed in triplicates.

As the bleached material is intended to be used as a fibre supplement in food products, it is important to understand how hydrogen peroxide bleaching affects the fibre content and composition. Therefore, three larger batches of Swe19 hulls were bleached with varying chemical loadings (75 kg H_2_O_2_/bdt and 5 kg NaOH/bdt; 100 kg H_2_O_2_/bdt and 10 kg NaOH/bdt; 150 kg H_2_O_2_/bdt and 10 kg NaOH/bdt; Procedure B). As shown in Fig. 6A, no significant differences in the carbohydrate content were found for untreated and bleached samples except for the starch content. Furthermore, the hemicellulose composition, which is mainly arabinoxylan (polymer consisting of mainly arabinose and xylose monomers), is not substantially affected either (see Fig. 6B).

Additionally, the amount of fibres found in the filtrate after bleaching (Filtrate I, see section 2.2) was analysed. Only very small amounts of fibres were found (0.02 % of treated hull fraction), supporting the finding that only minor amounts of fibres are lost during bleaching.

#### 3.1.5 Phenolics content

**Fig. 7:**
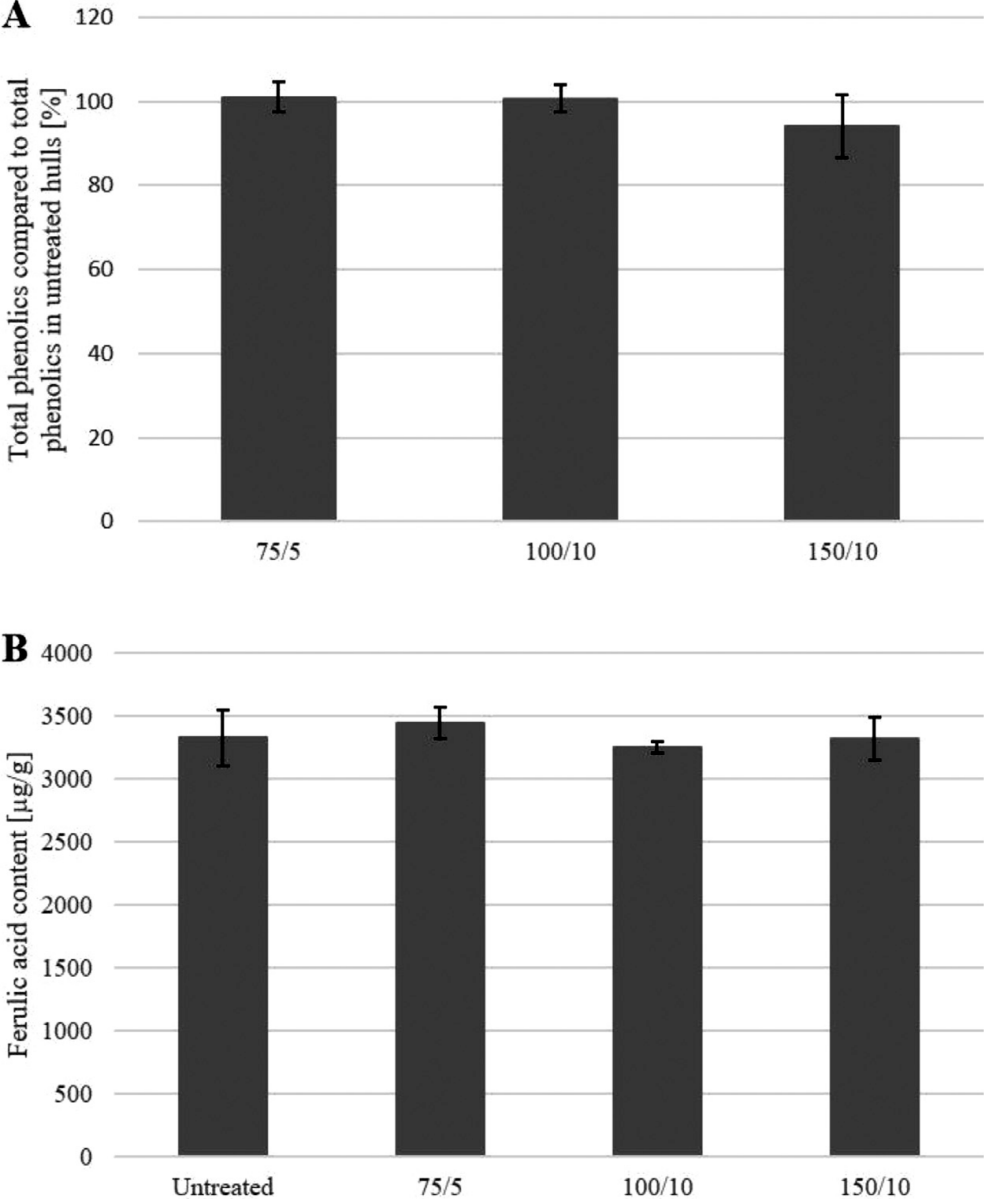
Total phenolics in bleached samples (Swe19) compared to untreated hulls (A) and total ferulic acid content in untreated and bleached hulls (B). The total phenolics content is reported as percentage based on the total phenolics content measured in an unbleached reference oat hull sample of the same batch. Bleaching conditions were 75 kg H_2_O_2_/bdt and 5 kg NaOH/bdt (denoted as 75/5 in the graphs), 100 kg H_2_O_2_/bdt and 10 kg NaOH/bdt (100/10) and 150 kg H_2_O_2_/bdt and 10 kg NaOH/bdt (150/10). All analyses were performed in triplicates.

Phenolics as well as lignin have been considered to significantly contribute to colour formation in biomass [5,21]. As the lignin composition was not affected to any large extent during bleaching and there is an exceptionally high presence of phenolic acids in oat hulls [1,22], the observed colour change might be due to a change in the phenolic acid composition. Therefore, the total phenolics content in the untreated and bleached Swe19 samples was analysed. As shown in Fig. 7, no difference in the phenolic acid content or composition (exemplified with ferulic acid content) was observed, proving that the low molecular weight phenolics are not affected by hydrogen peroxide bleaching. As neither the lignin is removed nor the phenolics composition changed, bleaching must occur via a lignin-retaining mechanism. Under such conditions, phenolic structures, especially the present hydroxyphenyl type phenolics, have been shown to be very stable [[23], [24], [25]].

**Fig. 8:**
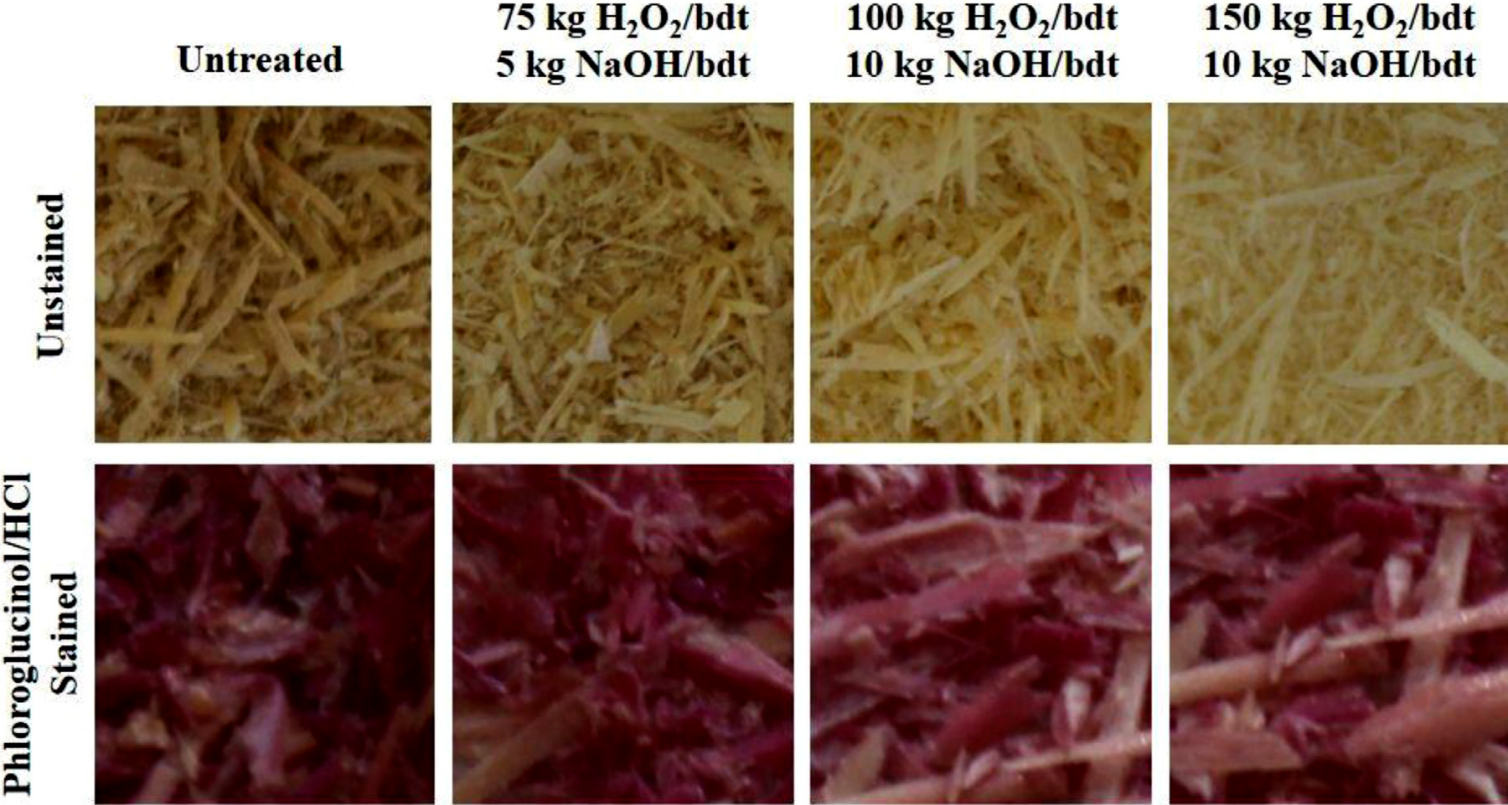
Visual appearance of the untreated and bleached oat hulls at different concentrations of bleaching chemicals before and after staining with phloroglucinol−HCl solution. The pink colour indicates the presence of coniferaldehyde structures.

Other strongly coloured species common in wood lignin are coniferaldehyde structures. Those are bound to the ends of the lignin polymers and hence, their side chains can easily be removed by an oxidative cleavage with bleaching chemicals resulting in less coloured α-carbonyl and hydroquinone structures. Pan and colleagues [24] have demonstrated that the bleaching effect of alkaline hydrogen peroxide on spruce thermomechanical pulp is mainly attributed to the removal of these structures. In order to analyse if these structures are also present in oat hull lignin and removed upon alkaline hydrogen peroxide bleaching, the Wiesner test was performed on unbleached and bleached oat hull samples. The Wiesner test involves a specific colour reaction of a phloroglucinol−HCl solution with coniferaldehyde structures. The dark pink colour of the unbleached oat hulls confirms the presence of coniferaldehydes (see Fig. 8). Alkaline hydrogen peroxide bleaching does lead to a reduction in the intensity of the pink colour indicating that some coniferaldehyde structures are removed. However, the strongest hydrogen peroxide bleached hulls are still stained by the Wiesner reagent showing that not all structures are removed. This is most likely due to the inaccessibility of those groups within the hull’s structure. Pan and colleagues [24] have shown that for a complete removal of all coniferaldehydes in spruce thermomechanical pulp, prior isolation of the lignin is required. Fig. 8 furthermore shows that the overall bleaching of the oat hulls seems to be more profound than the removal of coniferaldehyde structures. This suggests that other coloured species, potentially quinones, are removed upon bleaching.

These data show that chemical bleaching with alkaline hydrogen peroxide is a very suitable method for bleaching oat hulls intended for the use as fibre supplement in food products as a good brightness and lightness is reached, while the fibre composition is not substantially affected.

### 3.2 Biochemical/chemical bleaching of milled oat hulls

There are many reports in the literature suggesting the use of enzymes alone or in combination with chemicals for biomass bleaching [5,7,8]. Of particular interest for oat hull bleaching are laccases and xylanases in combination with sonication, as those have been shown to increase brightness in combination with chemical bleaching of wheat and rice straw, which have a similar chemical content compared to oat hulls [7]. In Ziaie-Shirkolaee et al. [7], an increase of up to 6.5 brightness points was obtained, which led to a possible reduction of the used bleaching chemical chlorine dioxide of up to 25 %. In order to successfully analyse the influence and interaction of the laccase, xylanase and sonication on the bleachability of oat hulls in a design of experiment study, a pre-study with laccase only was performed which aimed at determining the best framework conditions for the reaction.

#### 3.2.1 Laccase including treatments

**Fig. 9:**
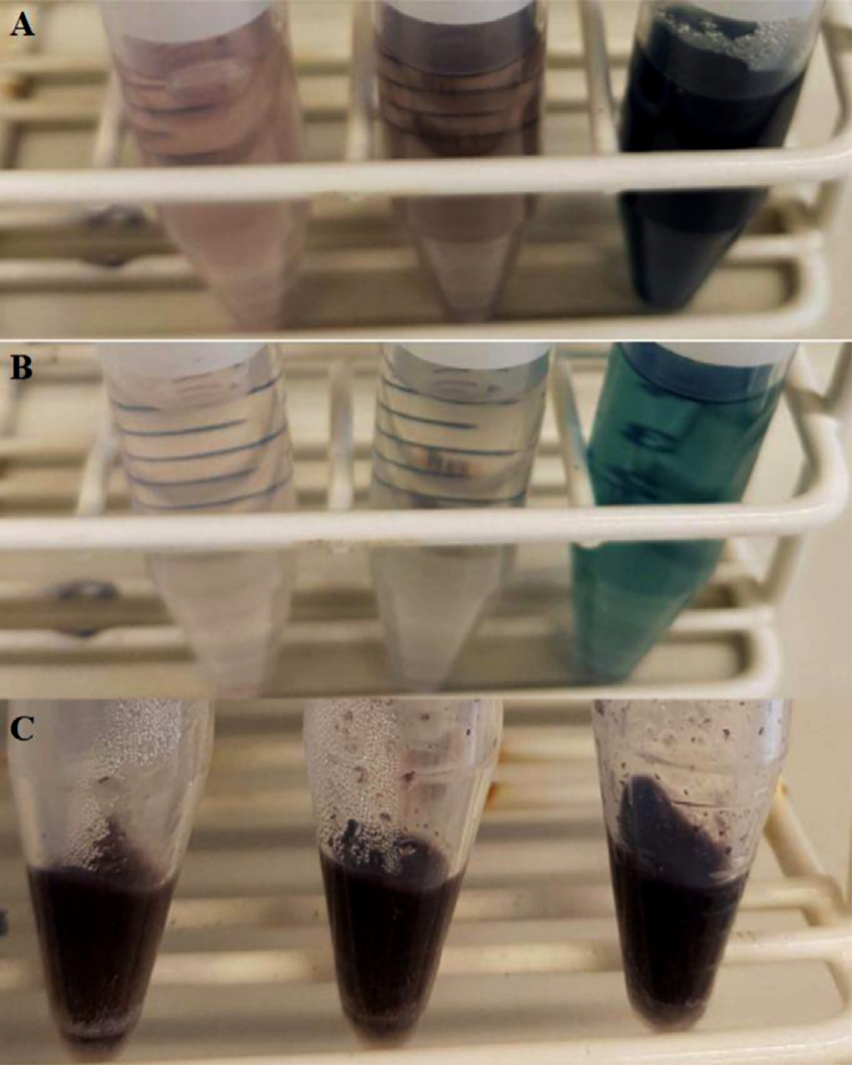
Colour of oat hulls (Swe18) treated with laccase and 0.5 mM, 1 mM and 3 mM ABTS (from left to right) at different wash stages: supernatant after the first water wash cycle (A), supernatant after the second wash cycle (B) and laccase-ABTS treated oat hulls after the second wash cycle (C).

Laccases have been shown to bleach better in presence of a mediator [5,8]. A commonly used one is 2,2′-azino-bis(3-ethylbenzothiazoline-6-sulfonic acid) (ABTS). However, the reaction of laccase and ABTS stained the hulls blue and it was not possible to wash (H_2_O) away the colour, even after two cycles (see Fig. 9). Therefore, ABTS is unsuitable for the purpose of bleaching oat hulls and no mediator was used in the following trials.

Another variable that could limit laccase activity is the availability of oxygen in the reaction medium. In order to assess the influence of this variable, the laccase reaction was performed with and without air sparging. The results show no difference in optical properties after a subsequent H_2_O_2_ bleaching stage (see Table 4). Hence, oxygen presence does not seem to be a limiting factor.

**Table 4:** The optical properties colour coordinates (L*, a*, b*) and brightness of untreated, laccase treated and laccase treated with air sparging, destarched and milled oat hulls (Swe18) followed by chemical bleaching and freeze drying. The conditions for chemical bleaching were 145 kg/bdt H_2_O_2_ and 10 kg/bdt NaOH at 70 °C, 120 min and a dry solids content of 35 %.

Sample	L*	a*	b*	Brightness [%]
Untreated + H_2_O_2_ bleached	84.8	−3.2	29.8	38.6
Laccase treated + H_2_O_2_ bleached	83.9	−3.2	29.7	37.3
Laccase treated with air sparging + H_2_O_2_ bleached	84.3	−3.2	29.5	38.0

While assessing the importance of air sparging, another important factor influencing hull colour was noted. When the hulls were oven dried (60 °C, overnight) after the enzymatic treatment, they became darker than the untreated ones. The formation of darker, insoluble compounds when oat hulls are subjected to higher temperatures has been observed before by Cardoso et al. [11], who suspected Maillard reactions and caramelization to be the cause. This darkening could not be reversed by subsequent chemical bleaching using H_2_O_2_/NaOH in the present study (see Table 5). Therefore, alternative freeze drying was tested. This drying method did not influence the hull colour and was therefore used for the trials described in Section 3.2.2.

**Table 5:** The optical properties colour coordinates (L*, a*, b*) and brightness of untreated, oven dried as well as freeze dried destarched and milled oat hulls (Swe18). All samples were chemically bleached with the following conditions: 145 kg/bdt H_2_O_2_ and 10 kg/bdt NaOH at 70 °C, 120 min and a dry solids content of 35 %.

Sample	L*	a*	b*	Brightness [%]
Untreated + H_2_O_2_ bleached	85.9	−2.5	31.3	38.9
Oven dried + H_2_O_2_ bleached	62.2	2.4	19.9	19.3
Freeze dried + H_2_O_2_ bleached	83.9	1.6	27.3	39.1

#### 3.2.2 Factorial design for enzymatic treatment and sonication

Based on the results of the pre-study, a design of experiment study was executed with varying concentrations of laccase and xylanase as well as sonication length. All treatments had a similar bleaching effect modifying the L* value slightly by -0.8 to 1.8 or -0.5 to 0.6 units for 50 or 100 kg H_2_O_2_/bdt, respectively (see Table 6). The best result was achieved for sample 5 as it shows the highest L* value at the lowest hydrogen peroxide loading. This sample has a long sonication time and low enzyme concentrations, indicating that sonication might be more important than enzymatic treatment. However, this difference is insignificant compared to only chemical bleaching. Furthermore, the achieved savings in bleaching chemical consumption are rather small. Considering that several extra processing steps are required for the enzymatic treatment including a milling and dewatering stage, this process would not be feasible on industrial scale. Therefore, it can be concluded that, using the enzymes selected here, the enzymatic treatments previously shown to be effective on other lignocellulosic materials, did not have the capability to bleach oat hulls. This is most likely due to the difference in recalcitrance of the different materials. The chemical composition of oat hulls suggests many ferulic acid ester linkages cross-linking the hemicellulose polymers to each other as well as to lignin [1]. This tight packing makes the material rather inaccessible to enzymatic attack. The lignocellulosic material previously bleached by biochemical methods was kraft pulp [7,8] and has hence been pre-treated with sodium sulphide and sodium hydroxide at high temperatures. This process leads to a dissolution and degradation of lignin making the hemicellulose more accessible to enzymatic attack [20].

**Table 6:** Factor settings and L* results for different H_2_O_2_ loadings of the design of experiment study using milled oat hulls (Swe19). The NaOH loading was 10 kg/bdt for all H_2_O_2_ loadings.

Sample	Sonication length [min]	Laccase concentration [U/g]	Xylanase concentration [U/g]	L* at H_2_O_2_, 50 [kg/bdt]	L* at H_2_O_2_, 100 [kg/bdt]	L* at H_2_O_2_, 150 [kg/bdt]
Ref^1^	---	---	---	78.1	82.5	86.9
1	10	6	5	79.3	83.1	84.0
2	10	18	5	79.5	82.8	83.8
3	10	6	15	79.3	82.0	84.1
4	10	18	15	78.3	82.1	83.9
5	60	6	5	79.9	82.6	83.8
6	60	18	5	79.3	83.1	83.6
7	60	6	15	77.3	82.7	83.9
8	60	18	15	77.5	82.0	83.9
9	35	12	10	79.3	82.6	83.8
10	35	12	10	77.8	83.1	83.8
11	35	12	10	78.6	82.5	84.3

1 Milled oat hulls (Swe19) that were heat treated (90 °C, 30 min) prior to H_2_O_2_ bleaching.

## 4 Conclusion

Chemical bleaching with hydrogen peroxide and sodium hydroxide is a suitable and robust method for bleaching of oat hulls intended to be used as dietary fibre supplements. Oat hull batch variability does not influence the required conditions for the desired optical properties very much despite variations in unbleached optical properties and chemical composition. Unmilled oat hulls were successfully bleached on the inside as well as outside, making it possible to simplify industrial processing by adding a milling step after the bleaching stage. Additionally, this type of chemical bleaching seems to be rather mild as the overall chemical composition was not substantially affected. Alkaline hydrogen peroxide is therefore an ideal bleaching option for retaining the oat hull composition that makes the bleached oat hull suitable as food ingredient.

In contrast, biochemical bleaching utilizing a xylanase, a laccase and sonication only had a minor positive effect on the L* value. A possible reduction of bleaching chemicals in a subsequent chemical bleaching step is, based on the current results, judged to be too small to be industrially feasible considering the extra processing steps required for biochemical bleaching. To implement this methodology in industry, more enzyme candidates should be tested. Elucidation of exactly which chemical structures are removed or modified in the chemical bleaching process is an interesting topic for future research. This could also give hints on which enzymes might give bleaching effects on oat hulls and similar materials.

## Author contributions

**Table:** 

**Contributor Role**	**Authors**
**Conceptualization**	Eva S, Eva NK, Patrick, Magnus
**Data Curation**	/
**Formal Analysis**	Eva S, Juanita, Katarina, Magnus
**Funding Acquisition**	Eva NK, Patrick, Magnus
**Investigation**	Eva S, Juanita, Katharina, Magnus
**Methodology**	Eva S, Juanita, Katharina, Magnus
**Project Administration**	Eva NK, Patrick, Magnus
**Resources**	/
**Software**	/
**Supervision**	Eva NK, Patrick, Magnus
**Validation**	Eva S, Juanita, Katharina, Magnus
**Visualization**	Eva S, Magnus
**Roles/Writing – Original Draft**	Eva S, Magnus
**Writing – Review & Editing**	All

## Role of funding source

This work was supported by the Swedish Governmental Agency for Innovation Systems (VINNOVA) [grant number 2017-02713] within the strategic innovation program BioInnovation, jointly organised by VINNOVA, The Swedish Research Council for Enviroment, Agricultural Sciences and Spatial Planning (Formas) and the 10.13039/501100004527Swedish Energy Agency. The award was received by ENK and PA. The funders had no role in study design, data collection, analysis and interpretation, decision to publish, or preparation of the manuscript.

## Declaration of Competing Interest

The authors declare that they have no known competing financial interests or personal relationships that could have appeared to influence the work reported in this paper.

Authors Magnus Paulsson and Katarina Gutke were employed by the company Nouryon. The remaining authors declare that the research was conducted in the absence of any commercial or financial relationships that could be construed as a potential conflict of interest.
